# Multiple Novel Alternative Splicing Forms of FBXW7α Have a Translational Modulatory Function and Show Specific Alteration in Human Cancer

**DOI:** 10.1371/journal.pone.0049453

**Published:** 2012-11-14

**Authors:** Yueyong Liu, Shancheng Ren, Andres Castellanos-Martin, Jesus Perez-Losada, Yong-Won Kwon, Yurong Huang, Zeran Wang, Mar Abad, Juan J. Cruz-Hernandez, Cesar A. Rodriguez, Yinghao Sun, Jian-Hua Mao

**Affiliations:** 1 Life Sciences Division, Lawrence Berkeley National Laboratory, Berkeley, California, United States of America; 2 Department of Urology, Changhai Hospital, Second Military Medical University, Shanghai, China; 3 Instituto de Biología Molecular y Celular del Cáncer (IBMCC), Instituto Mixto Universidad de Salamanca/CSIC, IBSAL, Campus Miguel de Unamuno s/n, Salamanca, Spain; 4 Deparment of Pathology, Hospital Universitario de Salamanca, IBSAL, Salamanca, Spain; 5 Department of Medical Oncology, Hospital Universitario de Salamanca, IBSAL, Salamanca, Spain; Indiana University School of Medicine, United States of America

## Abstract

*FBXW7* acts as a tumor suppressor through ubiquitination and degradation of multiple oncoproteins. Loss of FBXW7 expression, which could be partially attributed by the genomic deletion or mutation of FBXW7 locus, is frequently observed in various human cancers. However, the mechanisms regulating FBXW7 expression still remain poorly understood. Here we examined the 5′ region of *FBXW7* gene to investigate the regulation of FBXW7 expression. We identified seven alternative splicing (AS) 5′-UTR forms of FBXW7α that are composed of multiple novel non-coding exons. A significant difference in translational efficiency among these 5′-UTRs variants was observed by in vivo Luciferase reporter assay and Western blot. Furthermore, we found that the mRNA level of the AS form with high translational efficiency was specifically reduced in more than 80% of breast cancer cell lines and in more than 50% of human primary cancers from various tissues. In addition, we also identified mutations of FBXW7 in prostate cancers (5.6%), kidney cancers (16.7%), and bladder cancers (18.8%). Our results suggest that in addition to mutation, differential expression of FBXW7α AS forms with different translational properties may serve as a novel mechanism for inactivation of FBXW7 in human cancer.

## Introduction

The *FBXW7* gene is a transcriptional target of p53, whose expression is upregulated in a p53-dependent-manner after radiation treatment [1]. The *FBXW7* gene encodes an F-box protein, which is essential for the ubiquitination of different oncoproteins, including c-Myc [2], [3], c-Jun [4], cyclin E [5], [6], different members of the Notch family [7], [8], Aurora-A [1], [9], mTor [10], [11], and KLF5 [12], [13]. Overexpression of several of these targets, such as cyclin E [14], c-Myc [15] and Aurora-A [16] has been implicated to induce genomic instability. These observations demonstrated that *FBXW7* is a human tumor suppressor gene, a conclusion that is also supported by the discovery of *FBXW7* gene mutations in cancers from a wide spectrum of human tissues, such as bile duct, blood, bone, brain, breast, colon, endometrium, stomach, lung, ovary, pancreas, and prostate, with overall 6% point mutation frequency [17].

Deletion of the *Fbxw7* gene in mice leads to embryonic lethality, but heterozygous mice develop normally [18], [19]. Although they do not develop spontaneous tumors, radiation exposure gives rise to different types of tumors, including a range of epithelial cancers [1]. Mice that carry inactivated alleles of both *Fbxw7* and *p53* show acceleration of tumor development. Haploinsufficient loss of *Fbxw7* is observed in most lymphomas in this mouse model, even those arising from *Fbxw7/p53* double heterozygous mice, i.e., loss of only one copy of the gene can generate a substantial biological impact [1]. Similar observations of heterozygous mutations were subsequently found in human tumors [20]. It is therefore likely that the overall impact of this tumor suppressor gene in human cancer is greater than the 6% point mutation frequency mentioned above, since loss of only one gene copy can have a substantial effect on tumor development. Deletions of chromosome 4q31, on which *FBXW7* is located, are common in many types of human cancers [21]–[25], suggesting that disruption of this pathway may be a major feature of many, or even a majority, of human cancers.

The 5′ untranslated region (5′-UTR) plays an important role in the control of eukaryotic gene expression [26]. Recent studies on the mammalian transcriptome suggest that most of the genes express multiple alternative splicing (AS) 5′-UTRs, and UTR heterogeneity for a specific gene likely has a differential effect on protein expression [27], [28]. Notably, many oncogenes and tumors suppressor genes are also apt to express atypically complex 5′-UTRs [29], [30]. In addition, it is becoming clear that inappropriate expression of 5′-UTR AS has been shown to contribute to the development of cancer [31], [32].

In the present study, we investigated 5′ region of *FBXW7* to understand its regulatory mechanisms, and identified multiple novel non-coding exons in FBXW7α isoform, which produced multiple 5′-UTR AS forms. The functional impact of these 5′-UTRs on the efficiency of translation was shown to subsequently regulate FBXW7α expression. FBXW7α 5′-UTRs are differentially expressed between various normal and tumor tissues, which likely results in the change in the levels of FBXW7α expression during carcinogenesis. Our findings in this study suggest that differential expression of FBXW7α AS forms serves a new mechanism inactivating *FBXW7* in human cancer.

## Results

### Multiple novel alternative splicing forms identified in *FBXW7* gene

**Figure 1:**
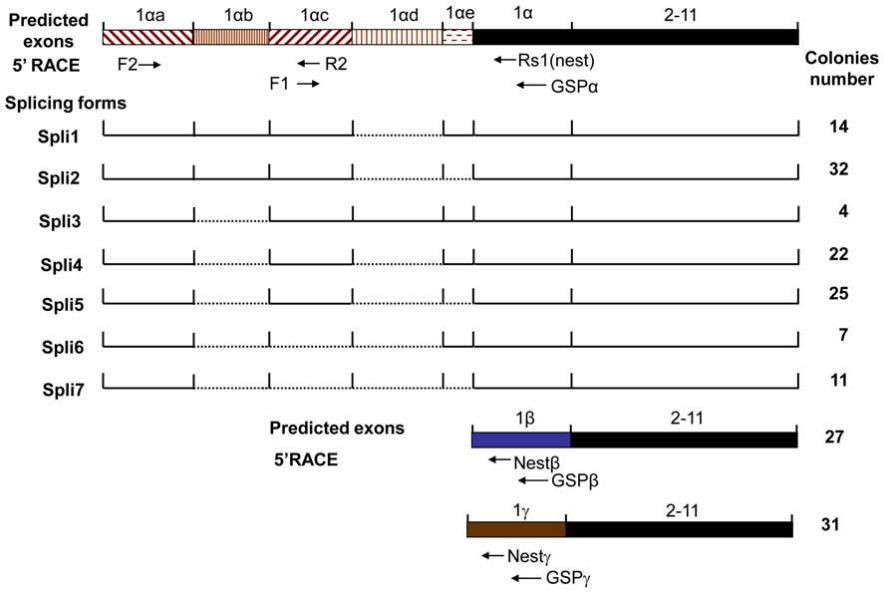
Characterization of N terminus of FBXW7 isoforms using 5′-RACE and bioinformatics analysis. Structural illustration of multiple splicing forms of FBXW7α. Seven different splicing forms of FBXW7α were identified by 5′-RACE using gene specific primer (GSP) and nest primer (Rs1). The number of colonies for each splicing form found during screening was shown. The N-terminal sequence of isoform β and γ was also investigated by 5′-RACE using the corresponding primers GSPβ, GSPγ, Nestβ and Nestγ. The arrows represent the primers used in this study and their position in the corresponding sequence.

Three FBXW7 isoforms (α, β and γ) have been reported so far. They differ in the first exon and share the following 2–11 exons. In order to examine the regulation of *FBXW7* expression, we characterized the 5′ region of FBXW7α, β and γ isoform using the 5′ RACE technique with cDNA from the human mammary epithelial cell (HMEC) 184A1. Sequencing analysis of 115 RACE clones revealed five novel non-coding exons within *FBXW7α* 5′-UTR when aligned with the human genomic sequence, whereas no additional exons were detected from *FBXW7β* and *FBXW7γ* by sequencing 27 and 31 RACE clones respectively (Figure 1). The *FBXW7α* 5′-UTR undergoes alternative splicing (AS) to produce seven mRNA transcripts with the same primary translation initiation site (Figure 1, Table S2). These results were further confirmed by blasting the sequences against the database of Expressed Sequence Tags (dbEST). The sequences of seven alternative splicing forms were deposited in Genebank with access number HQ873864-HQ873870.

Among these exons, 1αa and 1αc appear in most of AS forms, whereas 1αd is only in one out of seven splicing forms (Figure 1). Interestingly, 1αb and 1αd seem unlike to coexist in the same AS form. In order to confirm this, we designed specific PCR primers corresponding to the sequences in 1αb and 1αd, respectively (Figure S1) and were unable to detect any band from tissues mRNA used in this study by RT-PCR (data not shown). In addition, there are no new ORF found in all seven AS forms of FBXW7α, indicating that these new exons only form different 5′-UTR regions of different AS forms.

### Differences in the translation efficiency of different *FBXW7α* AS forms

**Figure 2:**
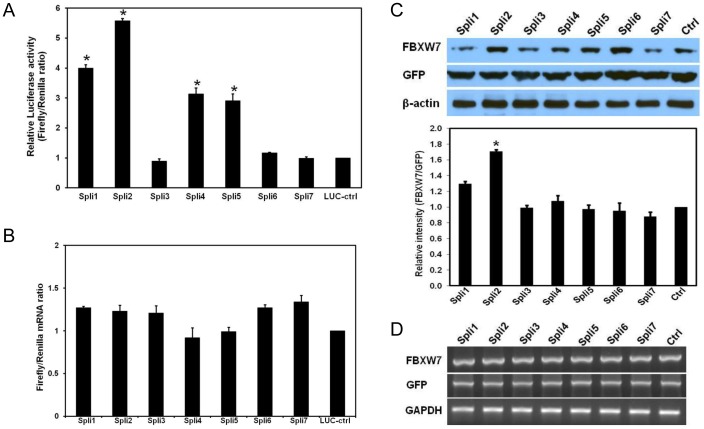
5′-UTRs of *FBXW7α* determine the translational efficiency. (A) Effect of *FBXW7α* 5′-UTR variants on LUC activity. LUC reporter constructs, which contained each *FBXW7α* 5′-UTR upstream of the LUC reporter gene in the pGL3 vector, were transfected into Hela cells. The LUC activity was measured as the Firefly/Renilla ratio. Results represent the data from three independent experiments. Each experiment was performed in triplicate. Mean data and standard deviations are shown. *p<0.05 in comparison to LUC-ctr. (B) LUC transcriptional levels were examined by qRT-PCR. The LUC mRNA contents were normalized to the Renilla mRNA contents for all samples and the relative LUC mRNA for pGL3p (empty vector, Promega) was arbitrarily considered to be 1 (control). (C) Effect of 5′-UTRs on the FBXW7α protein levels. Immunoblot was developed with anti-HA antibody from extracts with indicated construct. The intensity of pcDNA3.1(+) containing FBXW7α gene (control) was considered as 1. GFP construct was used as transfection efficiency control, and β–actin as loading control. All transfections were performed in triplicate, and the bars indicate the standard deviation. *p<0.05 in comparison to control. (D) cDNA frangments for FBXW7 (upper panel), GFP (middle panel) and GAPDH (lower panel) were specifically amplified by RT-PCR from Hela cells transfected with indicated constructs.

To investigate the functional effect of these 5′-UTRs on the translational efficiency of subsequent FBXW7α ORF, we first used a luciferase reporter assay to compare seven 5′-UTR sequences. To this end, we cloned each 5′-UTR upstream of the firefly luciferase (LUC) gene in the pGL3 promoter reporter vector (Promega), and transfected them into HeLa cells. Empty pGL3-promoter was used as a control (LUC-ctrl). Renilla-normalized luciferase activity for each construct was compared with the LUC-ctrl. The results of these assays consistently showed the significant differences in LUC activities among these seven 5′-UTR variants (Figure 2A). Spli2-UTR always exhibited the highest LUC activity while the level of LUC activity in Spli1-UTR, Spli4-UTR, and Spli5-UTR was intermediate, but significantly higher than LUC-ctrl; the LUC activity of Spli3-UTR, Spli6-UTR, and Spli7-UTR was not statistically significant in comparison with LUC-ctrl (Figure 2A). In order to corroborate that these differences in LUC activity were not due to the variations in LUC transcription, we performed quantitative real time RT-PCR (qRT-PCR). As showed in Figure 2B, there were no significant differences in the levels of LUC transcription among different 5′-UTR variants when compared to LUC-ctrl. This result clearly indicates that the 5′-UTRs of FBXW7α regulates the translational efficiency of downstream ORFs.

To further verify the impact of 5′-UTRs on the regulation of translation, we cloned seven 5′-UTRs into upstream of the FBXW7α ORF in pcDNA3.1 tagged with HA epitope and subsequently transfected them into Hela cells. Western blotting analysis showed expression level from the Spli2 construct was consistently higher than that observed with the other UTRs and control constructs when normalized to the level of transfection efficiency control GFP protein (Figure 2C), which is consistent with the results from the luciferase reporter assay. We confirmed that the expression difference is not due to transcriptional effects since mRNA levels are not significantly different (Figure 2D). Taken together, we concluded that the 5′-UTR variants of FBXW7α have translational modulatory function.

### Expression profiles of *FBXW7α* AS forms in human normal tissues

**Figure 3:**
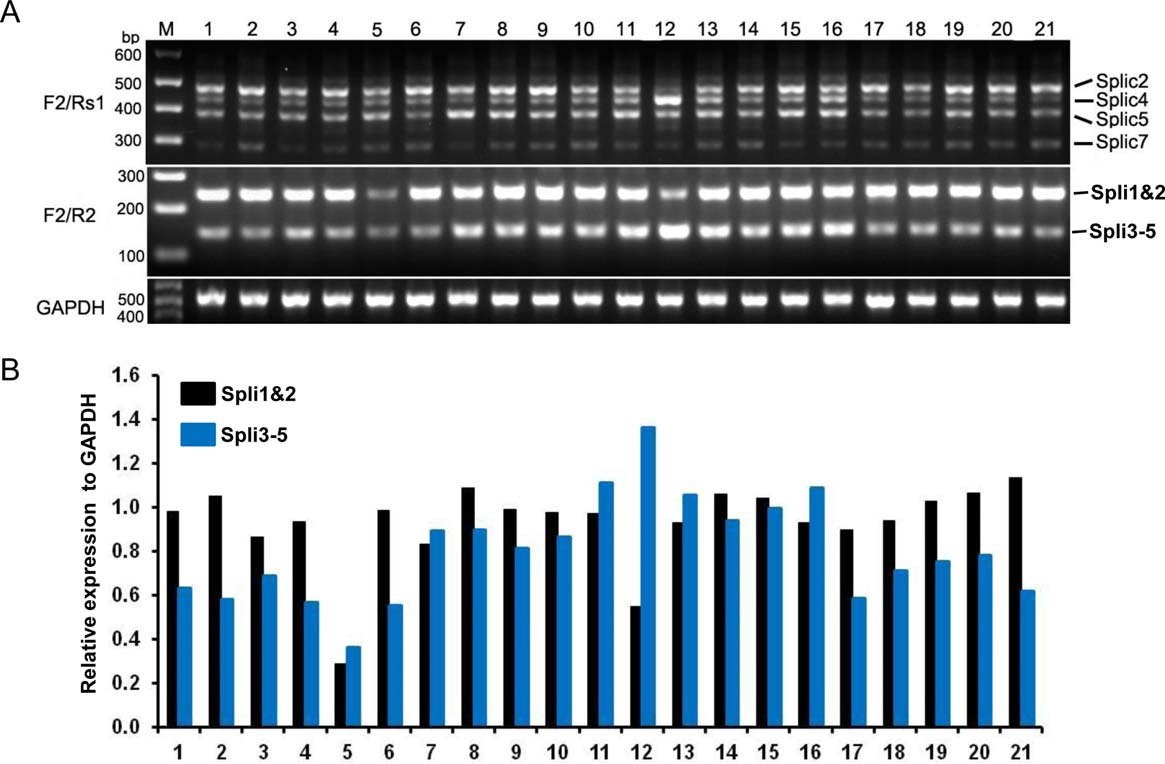
mRNA expression profile of *FBXW7α* AS forms in human normal tissues. (A) *FBXW7α* AS forms expression levels were detected by semi-quantitative RT-PCR using different pairs of primers. (B) The levels of Spli1&2 and Spli3-5 expression were quantified from the experiment shown in (A) lower panel. Normal tissues include: 1-esophagus, 2-adipose, 3-heart, 4-bladder, 5-kidney, 6-brain, 7-liver, 8-lung, 9-cervix, 10-colon, 11-spleen, 12-testes, 13-thymus, 14-thyraoid, 15-trachea, 16-small intestine, 17-skeletal muscle, 18-prostate, 19-placental, 20-ovary, 21-breast. “M” represents DNAs ladder Marker.

Given that 5′-UTR can regulate the translation of downstream ORFs, we next sought to investigate the expression pattern of *FBXW7α* AS forms in human tissues. For this purpose, semi-quantity RT-PCR was performed on 21 human normal tissues with different sets of primers corresponding to newly identified exons (Figure 1, Table S1). RT-PCR was initially performed with a pair of primers (F2/Rs1) that could detect all AS forms. As expected, multiple AS forms were expressed in all human tissues (Figure 3A, upper panel). The AS form Spli5, Spli4 and Spli2, corresponding to band sizes 386 bp, 435 bp, 478 bp respectively, showed high level of mRNA expression (Figure 3A, upper panel), consistent with our findings in 5′ RACE studies, where the number of clones corresponding to these AS forms were found much higher frequency (Figure 1). Expression level of different FBXW7α AS forms varied among tissues. For example, the Spli4 is the dominant form in testes (lane 12 in Figure 3A, upper panel) whereas the Spli2 is the most expressed one in other tissues (Figure 3A, upper panel), suggesting a tissue-associated distribution pattern.

As the seven AS forms contain crossover of several exons in 5′-UTR, it is difficult to distinguish these variants. Thus we designed the primers F2/R2 located in exon 1αa and 1αc to compare the expression level of the Spli1&2 (a sum of Spli1 and Spli2 expression) with the forms Spli3-5 (a sum of Spli3, Spli4 and Spli5 expression). We found that Spli1&2 predominantly expressed in most tissues (Figure 3A, lower panel, and Figure 3B). But some tissues, such as spleen, thymus and small intestine showed equal expression levels of Spli1&2 and Spli3-5 (lanes 11, 13 and 16 in Figure 3A, lower panel, and Figure 3B), while Spli3-5 is only high expressed in testes (lane 12 in Figure 3A, lower panel, and Figure 3B).

### Alternated expression of *FBXW7α* specific AS forms in human cancers

**Figure 4:**
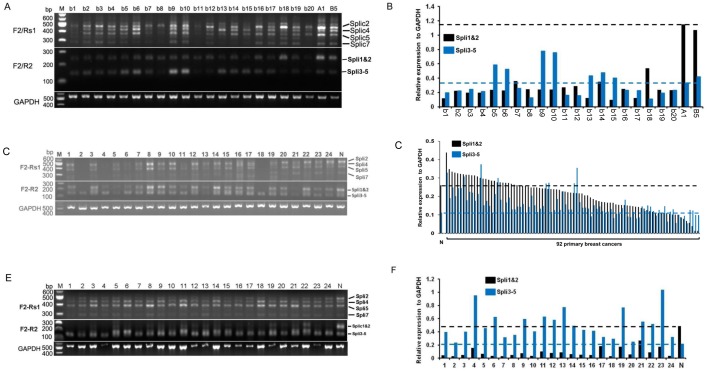
Differential change in mRNA expression level of FBXW7α AS forms in human cancer. (A) mRNA expression profile of FBXW7α AS forms in human breast cancer and HMEC cell lines was determined by semi-quantitative RT-PCR using different sets of primers. “b1 to b20” represents 20 different breast cancer cell lines, “A1” stands for 184A1, “B5” for 184B5. (B) The levels of Spli1&2 and Spli3-5 expression were quantified from the experiment shown in (A) lower panel. (C) The representative mRNA expression profile of FBXW7α AS forms from 92 human primary breast cancers by semi-quantitative RT-PCR using different sets of primers. (D) Quantification of Spli1&2 and Spli3-5 expression levels in 92 human primary breast cancers. (E) The mRNA expression profile of FBXW7α AS forms in 24 human primary kidney cancers by semi-quantitative RT-PCR using different sets of primers. (F) Quantification of Spli1&2 and Spli3-5 expression levels in 24 human primary kidney cancers. “N” for pooled RNAs from normal tissues, “M” for DNAs ladder Marker.

Since the differential expression of 5′-UTR AS forms has been linked with tumor progression, we investigated the expression profile of the identified FBXW7α AS forms in human cancer by semi-quantitative RT-PCR using the same set of primers described above. The result in a panel of 20 breast cancer cell lines showed that most breast cancer cell lines have decreased expression levels of FBXW7α specific AS forms compared with normal human mammary epithelial cells (HMEC) (184A1 and 184B5) (Figure 4A). Almost all breast cancer cell lines showed remarkable reduction in the expression of Spli1&2 whereas there are no changes in the expression of Spli3-5 compared to the levels in HMECs (Figure 4A, lower panel, Figure 4B). These observations were further confirmed in a set of 92 human primary breast cancers. In comparison to the pooled normal breast tissues, Spli1&2 expression levels showed a significant reduction in more than 50% of tumors. Surprisingly, we found that the Spli3-5 expression levels showed significant increase in more than 30% of tumors (Figure 4C and D).

Next we determined whether differential expression of *FBXW7α* specific AS also occurred in other types of human cancer. Indeed, the *FBXW7α* expression is switched from Spli1&2 to Spli3-5 in human kidney, prostate and bladder cancers (Figure 4E and F, Figure S2A and S2B). This differential expression pattern of FBXW7α AS forms in various human cancers substantially supports our hypothesis that FBXW7α AS forms involve in the regulation of FBXW7α during tumorigenesis. Together, our results suggest that total FBXW7 protein levels may be reduced in tumor cells through differential expression of FBXW7α AS forms, and the decrease in FBXW7 abundance substantially affects its function on ubiquitination and degradation of its targets (oncoproteins), consequently resulting in tumor development and progression.

### Mutational analysis of *FBXW7* in human urological cancers

**Figure 5:**
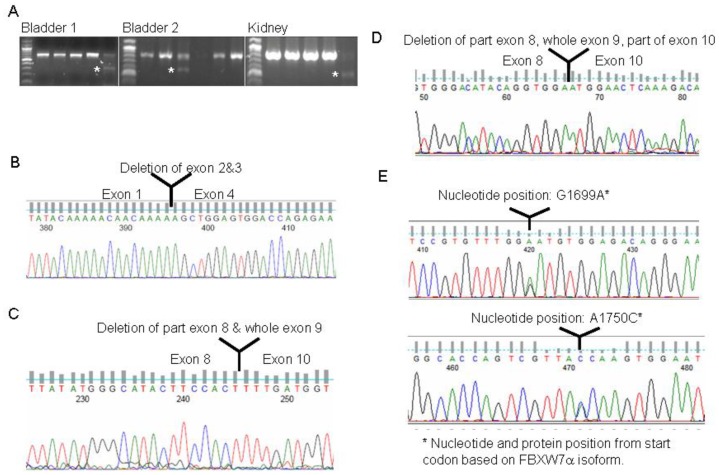
Mutational analysis of *FBXW7* in primary prostate, bladder, and kidney cancers from the Chinese patients. (A) RT-PCR analysis of *FBXW7*, RT–PCR products containing deletions are indicated by asterisk. (B–D) sequencing confirmed the deletion in two bladder cancers (B and C), and a kidney cancer (D). (E) Representative sequence traces showing the mutations of *FBXW7*.

Although mutations are rarely detected in breast cancers, genetic alterations are still found in prostate cancers [35], and there is no report about the genetic alterations in bladder and kidney tumors. Additionally, cancer disparities have been found among different ethnic groups. There is also no report concerning the *FBXW7* mutation spectrum in cancer among Chinese patients. This intrigues us to examine whether there are mutations and what is the frequency of the mutations in Chinese cancer patients. Thus, we performed the mutation analysis of *FBXW7* gene in 18 prostate, 24 kidney, and 16 bladder tumor tissues from Chinese patients. Both mutations and deletions were found in these tumors as shown by either gel electrophoresis or sequencing map in Figure 5. Sequencing of shortened RT-PCR fragments confirmed two bladder samples and one kidney sample have deletions. Of the bladder tumor deletions, one tumor has a deletion of part of the exon 8 plus the whole exon 9, while in the other bladder tumor the whole exon 2 and 3 sequences are missing. The kidney cancer has a deletion of part of the exons 8 and 10 together with the whole exon 9 (Figure 5B–D, Table 1). The overall mutation rate of FBXW7 in prostate cancers is 5.6%, 16.7% in kidney cancers, and 18.8% in bladder cancers from Chinese patients as summarized in Table 1.

**Table 1:** Mutations in *FBXW7* gene in urological cancer from Chinese patients.

Tumor type	Frequency	Nucleotide or deletion (cDNA)*	Amino acid (protein)
Prostate	1/18	C383T	S128T
Kidney	4/24	T1453C	V485A
		G1699A#	D567N
		A1750C#	T584P
		A1792G	N598D
		del1270–1758 (part of exon8, exon9, and part of exon10)	
Bladder	3/16	A492C	K164N
		del502–727 (exon 2&3)	
		del1390–1644 (part of exon 8, and exon 9)	

* Nucleotide and protein position from the start codon based on FBXW7α isoform.

# two mutations from one sample.

## Discussion

This study provides new insights into the mechanisms that regulate FBXW7α expression through a novel splicing pattern. We have identified seven novel AS forms of *FBXW7α* by 5′ RACE technique. Although they produce essentially the same protein, the *FBXW7α* AS forms appear to control protein expression via the regulation of translational efficiency of these AS forms since we have demonstrated that FBXW7α 5′-UTR variants have a translational modulatory function. Multiple AS forms were only found in *FBXW7α*, not in *FBXW7β* or *FBXW7γ*. Nevertheless, we found that *FBXW7*α dominantly expressed in almost all of human tissues examined whereas *FBXW7*β mRNA is enriched in brain and thymus and is absent or in trace amount in other tissues, and *FBXW7*γ mRNA is found to be restricted in heart and skeletal muscle (Figure S3), which highlights the potential importance of *FBXW7*α to the function of FBXW7. Thus the multiple AS forms presented in FBXW7α may allow the precise regulation of its expression during biological processes.

We for the first time discovered that the protein levels of FBXW7α are regulated through translational control by demonstrating the differential translational efficiency of different FBXW7α AS forms. The *in vivo* experiments using LUC reporter assays consistently showed that the Spli2 form has the highest translational efficiency in comparison to others. Several factors are known to determine translational efficiency [33]. The differences in translational efficiency among FBXW7α 5′-UTR variants is unlikely due to their length, the sequence around translation start site, and number of start codons within them as the same number of start codons and the same sequence around translation start site were found in all FBXW7α 5′-UTR variants. The length of Spli2-UTR is clearly longer than the Spli6-UTR and Spli7-UTR, shorter than Spli3-UTR, but Spli2 exhibited higher translation efficiency. In addition, we examined differences in secondary structures and free energy of each *FBXW7α* 5′-UTR variants using the online RNA-folding software (http://rna.tbi.univie.ac.at/cgi-bin/RNAfold.cgi.). There are no significant differences in the free energy value after normalizing to the length among these 5′-UTRs (data not shown). Future studies will be granted to investigate the potential mechanisms by which 5′-UTRs regulate the translational efficiency.

*FBXW7* has emerged as a major human tumor suppressor gene. Several mechanisms have been reported for inactivation of FBXW7 in human cancer including mutation, deletion and hypermethylation [17], [21]–[25], [34]. A lot of efforts have focused on the finding of FBXW7 mutation in various types of human cancer and have shown that the overall point mutation frequency is only 6% in human cancers [17], but there was substantial variation across tumor types. Approximately 30% mutation frequency was found in cholangiocarcinoma and T-cell acute lymphoblastic leukamias whereas mutation frequencies in prostate, endometrial cancers as well as gastrointestinal cancer arrange from 4% to 15% [35]. Consist with these findings, we reported here for the first time the mutations of FBXW7 detected in human kidney and bladder tumors with the frequency 16.7% (4/24) and 18.8% (3/16) (Table 1), respectively. The frequency in prostate tumor from Chinese population is 5.6%, similar to a previous study carried out in Caucasians [36].

Most important finding in this study is that the *FBXW7* gene is deregulated through differential expression of AS forms in human cancers. The connection between tumor development and deregulation of AS have become a novel and important aspect of cancer biology. Our results show that the transcriptional pattern of *FBXW7* AS forms in human cancers is different from normal tissue compartment. As shown in Figure 4, the *FBXW7α* expression patterns in the majority of human cancers are switched from Spli1&2 to Spli3-5 in comparison to their normal tissue. The consequence of this switch causes the reduction of *FBXW7* protein level, and in turn leads to partial loss of its function. This result suggests that deregulated AS of *FBXW7α* gene may be an additional mechanism to inactivate *FBXW7* in human cancers. Since differential expression of *FBXW7α* AS forms has been found in more than 50% of cancers, we proposed that this new mechanism plays a major role in human cancer development.

In conclusion, by analyzing *FBXW7* 5′ end region, we identified the novel *FBXW7α* 5′-UTR variants that regulate *FBXW7α* gene expression through the mechanism involving translational efficiency. These AS forms are expressed in a tissue-associated manner with varied levels among different tissues. Our study provides a new insight into the alteration of *FBXW7α* expression during human cancer development.

## Materials and Methods

### Ethics statement

The human primary breast tumors were collected at the time of surgical resection at Hospital Universitario of Salamanca, Salamanca, Spain. Collection and the use of patient samples were approved by the institutional ethics review board of the Hospital Universitario of Salamanca. The human primary prostate, bladder and kidney tumors were collected at the time of surgical resection at Changhai Hospital, China. The collection and the use of patient samples were approved under the institutional ethics review board of Changhai Hospital, Second Military Medical University. Written informed consent was obtained from all patients for research using these tumor samples.

### Patient samples

In both hospitals, the fresh tumor tissue samples were obtained at the time of surgical resection of patient tumors. The samples were immediately snap-frozen down in liquid nitrogen and then storage at −80°C freezer before use. H&E (hematoxylin and eosin stained) slides of frozen human tumor tissues were examined by the pathologists of this study to ensure that the tumor tissues selected had high-density cancer foci (>80%).

### Cell lines

Breast cancer cell lines were cultured in DMEM or in RMPI1640 supplemented with 10% fetal bovine serum and 100 mM Streptomycin and Penicillin; details were described in our previous study [37]. Hela cells were cultured in MEM with 10% fetal bovine serum and 100 mM Streptomycin and Penicillin. Cells were incubated at 37°C in a humidified incubator with 5% CO_2_.

### Total RNA extraction from cells and tissue

Total RNAs were prepared from 80% confluent culture of breast cancer cell lines, Hela cells 36 hr post transfection and frozen human breast, kidney, prostate and bladder tumor tissues using Trizol (Invitrogen) according to the manufacturer's instructions. Extracted RNA samples were treated with the DNA-free kit (Ambion) prior to further analysis, in order to remove any residual amount of genomic DNA. RNA concentration and quality were determined with a Nanovue spectrophotometer (GE healthcare). Human total RNA from different normal tissues was purchased from Ambion.

### 5′ rapid amplification of cDNA ends (RACE)

The characterization of *FBXW7* gene N terminus was carried out using 5′ RACE kit (Invitrogen) according to the manufacturer's instructions. Briefly, total RNA was isolated from the human mammary epithelial cell (HMEC) 184A1 cells with Trizol (Invitrogen), and cDNA was generated using specific primer (GSP) listed in Table S1. The cDNA was tailed with dCTP using terminal deoxy-transferase, and a poly-G primer (Invitrogen) was combined with each *FBXW7* cDNA for PCR. A second PCR was carried out with a dilution of the first PCR, using each nest primer Nestα (Rs1), Nestβ or Nestγ (Table S1), and an adaptamer primer provided by Invitrogen. The PCR products were analyzed by gel electrophoresis and were cloned into pCR4 vector. Each clone was sequenced.

### Reverse transcription and PCR amplification of cDNA

First strand cDNA was synthesized using SuperScript III cDNA synthesis kit (Invitrogen). 1 µg of RNA was subjected to reverse transcription following the reaction conditions specified by the manufacture. Reactions were primed with 50 ng of random hexamers. The cDNAs from breast cancer cell lines, human normal tissues panel, and primary breast, kidney, prostate and bladder tumors were analyzed for AS transcripts of *FBXW7*α using PCR with primer pairs F2/RS1, F2/R2 and F1/Rs1 (Table S1). The housekeeper gene GAPDH was amplified as control by the primer pair GapF/GapR (Table S1). The protocol for the PCR reaction was 26–35 cycles of denaturation at 95°C for 30 s, annealing at 56°C for 30 s, and extension at 72°C for 30 s.

To detect FBXW7 mutations in primary prostate, bladder, and kidney tumors, the coding sequence of *FBXW7* was amplified by RT-PCR using three primer pairs P1F/R, P2F/R and P3F/R (Table S1), and the amplified products were sequenced and compared in the Genebank Database. For different sized PCR products, bands were gel excised and purified, and then verified by DNA sequencing.

### Plasmid constructs

Seven different FBXW7α 5′-UTRs (Spli1 to 7) were amplified from those positive pCR4 clones in RACE using the following primer pair GL3F/GL3R (Table S1). The PCR products were gel-purified, digested and then ligated into pGL3-promoter vector (Promega) via HindIII/NcoI restriction sites immediately adjacent to the downstream luciferase gene. The generated constructs were designated as Spli1-UTR, Spli2-UTR, Spli3-UTR, Spli4-UTR, Spli5-UTR, Spli6-UTR, and Spli7-UTR. Similarly, seven 5′-UTRs amplified by primer pair GL3F/GL3R2 were introduced into upstream of FBXW7α gene fused with HA epitope in pcDNA3.1 (+) vector (saved in this laboratory) via HindIII/EcoRI. All constructs were verified by DNA sequencing.

### Transient transfection

Transient transfections were performed on about 70% confluent HeLa cells using the Lipofectamine 2000 reagent (Invitrogen). Twenty-four hours before transfection, cells were trypsinized and transferred to either 96-well plates (for luciferase assay) or 60 mm dishes (for RNA isolation). Transfection was performed with the reagent following the manufacture's protocol. Briefly, 0.2 µg of experimental DNA from large-scale plasmid preparations was delivered to the each well in the 96-well plates in the presence of phRL-TK *Renilla* luciferase control plasmid (Promega, 0.01 µg) or 4 ug of experimental DNA and 0.2 µg of *Renilla* luciferase control plasmid to the cells seeded in 60 mm dish, and cells were incubated at 37°C. After 4 hours, medium was replaced by DMEM with 10% FBS and antibiotics/antimycotics, and cells were incubated until used for RNA extraction or luciferase assay. After 6 hours, medium was replaced by DMEM with 10% FBS, and cells were incubated until used for RNA extraction or luciferase assay.

### Wesstern blot

Hela cells were co-transfected by ten micrograms each of pcDNA3.1containing alternative splicing form and pEGFP-C1 (Promega) for 48 hr and harvested by RIPA buffer (sigma). Fifty micrograms of protein was resolved on an 4–12% gradient SDS-polyacrylamide gel (Invitrogen) and electrotransfered to a PVDF membrane. Antibodies used in this study were anti-HA antibody (Sigma, clone HA-7), anti-GFP (cell signaling) and anti-beta-actin (Sigma, clone AC-15), and goat anti-mouse HRP-conjugated secondary antibody. Blots were developed with SuperSignal substrate (Thermo Scientific) and quantified using ImageJ software.

### Luciferase activity assay

Transfected cells were harvested after 36 hours, and both Firefly and Renilla luciferase activities were measured with the Dual-luciferase reporter assay system kit (Promega) according to the manufacturer's protocol using Modulus II microplate multimode reader (Turner Biosystem). Briefly, the cells were rinsed twice with 1× PBS and lysed in 40 µl of 1× passive lysis buffer (PLB) following gently rocking for 15 min at room temperature. For Firefly luciferase activity, 100 µl of LARII (luciferase assay reagent II) was added to each well and mixed by slightly tapping the plate. Place the plate in the luminometer and initiate reading. And then add 100 µl of Stop and Glo reagent to stop Firefly luciferase activity and simultaneously activate the Renilla luciferase activity, then place the plate in the luminometer for reading. Results are expressed by the ratio of Firefly to Renilla luciferase activities.

### Measurement of luciferase mRNA level using real-time RT-PCR

The amount of luciferase mRNA was determined by real-time RT-PCR using the Bio-rad CFX96 Real-Time PCR system and the Power SYBR Green Master mix (Applied Biosystems), according to the manufacturer's instructions. Renila luciferase was used as an internal control. The PCR conditions were 95°C for 10 min, and 40 cycles of 95°C for 15 s and 60°C for 1 min and 95°C for 1 min. The primers used for luciferase were LucF and LucR, and for Renila luciferase are ReniF and ReniR (Table S1).

### Statistical analysis

At least three independent experiments were carried out for transfection, mRNA quantification and luciferase assays. Differences among treatment groups were estimated by one-way ANOVA followed by the Tukey's multiple comparisons test using the Originlab7.5 software. The results are expressed as means ± standard deviation. Statistically significant differences were considered when p<0.05.

## Supporting Information

Figure S1 Genomic structure of FBXW7α. 1αa, 1αc, 1αb and 1αe are additional exons identified in this study.
(DOC)

Figure S2 Differential change in mRNA expression level of FBXW7α AS forms in human prostate (A) and bladder (B) cancers. The mRNA expression profile of FBXW7α AS forms was determined by semi-quantitative RT-PCR. “M” for DNAs ladder Marker.
(DOC)

Figure S3 The mRNA levels of Fbxw7α, Fbxw7β and Fbxw7γ in the different tissues were determined by RT-PCR analysis using corresponding primer pairs (Forward primers: Fα, Fβ, Fγ, and common reverse primer cdc4R) listed in Table S1. Normal tissues include: 1-esophagus, 2-adipose, 3-heart, 4-bladder, 5-kidney, 6-brain, 7-liver, 8-lung, 9-cervix, 10-colon, 11-spleen, 12-testes, 13-thymus, 14-thyraoid, 15-trachea, 16-small intestine, 17-skeletal muscle, 18-prostate, 19-placental, 20-ovary, 21-breast. “M” represents DNAs ladder Marker.
(DOC)

Table S1 The primers used in this study.
(DOC)

Table S2 The sequences of each FBXW7a 5′-UTR AS form.
(DOC)
