# Supplementary figures and images for: Multiple Novel Alternative Splicing Forms of FBXW7α Have a Translational Modulatory Function and Show Specific Alteration in Human Cancer
Source: PLoS One. 2012 Nov 14;7(11):e49453. doi: 10.1371/journal.pone.0049453 (PMC3498124; doi:10.1371/journal.pone.0049453)

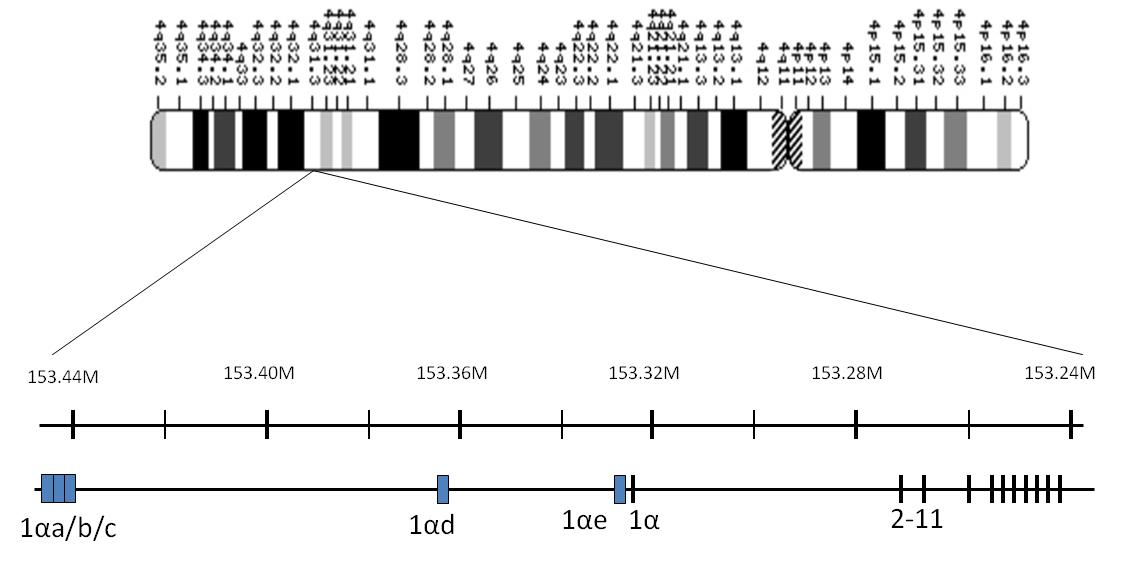


**Figure S1.** Genomic structure of FBXW7 1αa, 1αc, 1αb and 1αe are additional exons identified in this study.

Supplement: Figure S1 — Genomic structure of FBXW7α. 1αa, 1αc, 1αb and 1αe are additional exons identified in this study. (DOC) [file pone.0049453.s001.doc]
