# Supplementary material for: Multiple Novel Alternative Splicing Forms of FBXW7α Have a Translational Modulatory Function and Show Specific Alteration in Human Cancer
Source: PLoS One. 2012 Nov 14;7(11):e49453. doi: 10.1371/journal.pone.0049453 (PMC3498124; doi:10.1371/journal.pone.0049453)
Supplement: Figure S2 — Differential change in mRNA expression level of FBXW7α AS forms in human prostate (A) and bladder (B) cancers. The mRNA expression profile of FBXW7α AS forms was determined by semi-quantitative RT-PCR. “M” for DNAs ladder Marker. (DOC) [file pone.0049453.s002.doc]

**
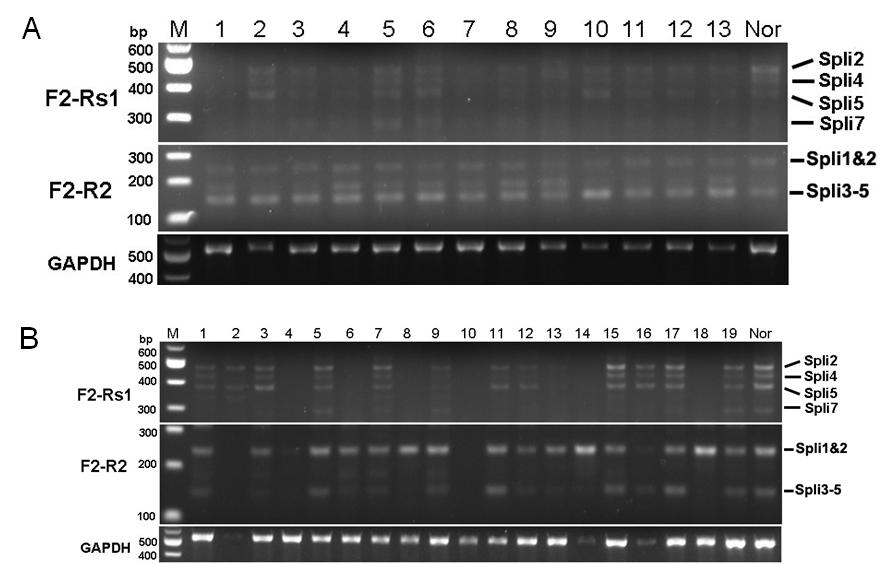
**

**Figure S2.** Differential change in mRNA expression level of FBXW7α AS forms in human prostate (A) and bladder (B) cancers. The mRNA expression profile of FBXW7α AS forms was determined by semi-quantitative RT-PCR. “M” for DNAs ladder Marker.
