# Supplementary material for: Multiple Novel Alternative Splicing Forms of FBXW7α Have a Translational Modulatory Function and Show Specific Alteration in Human Cancer
Source: PLoS One. 2012 Nov 14;7(11):e49453. doi: 10.1371/journal.pone.0049453 (PMC3498124; doi:10.1371/journal.pone.0049453)
Supplement: Figure S3 — The mRNA levels of Fbxw7α, Fbxw7β and Fbxw7γ in the different tissues were determined by RT-PCR analysis using corresponding primer pairs (Forward primers: Fα, Fβ, Fγ, and common reverse primer cdc4R) listed in Table S1. Normal tissues include: 1-esophagus, 2-adipose, 3-heart, 4-bladder, 5-kidney, 6-brain, 7-liver, 8-lung, 9-cervix, 10-colon, 11-spleen, 12-testes, 13-thymus, 14-thyraoid, 15-trachea, 16-small intestine, 17-skeletal muscle, 18-prostate, 19-placental, 20-ovary, 21-breast. “M” represents DNAs ladder Marker. (DOC) [file pone.0049453.s003.doc]

**
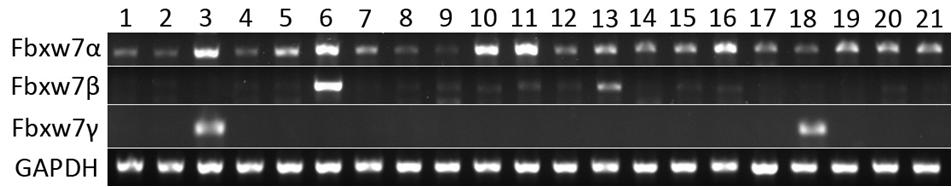
**

**Figure S3.** The mRNA levels of Fbxw7α, Fbxw7β and Fbxw7γ in the different tissues were determined by RT-PCR analysis using corresponding primer pairs (Forward primers: Fα, Fβ, Fγ, and common reverse primer cdc4R) listed in Table S1. Normal tissues include: 1-esophagus, 2-adipose, 3-heart, 4-bladder, 5-kidney, 6-brain, 7-liver, 8-lung, 9-cervix, 10-colon, 11-spleen, 12-testes, 13-thymus, 14-thyraoid, 15-trachea, 16-small intestine, 17-skeletal muscle, 18-prostate, 19-placental, 20-ovary, 21-breast. “M” represents DNAs ladder Marker.
