# Supplementary material for: Multiple Novel Alternative Splicing Forms of FBXW7α Have a Translational Modulatory Function and Show Specific Alteration in Human Cancer
Source: PLoS One. 2012 Nov 14;7(11):e49453. doi: 10.1371/journal.pone.0049453 (PMC3498124; doi:10.1371/journal.pone.0049453)
Supplement: Table S1 — The primers used in this study. (DOC) [file pone.0049453.s004.doc]

**Table S1. The primers used in this study.**

| Primers for 5’ RACE | | |
| --- | --- | --- |
| Isoform | Gene specific primer (GSP, 5'-3') | Nest primer ( 5'-3') |
| Fbxw7 α | CTGTCCTTGCTGGGAATCAT | TGAGTTGCTGTTGCTGTTCC |
| Fbxw7 β | GGCACGTCAGAAAAGGAAGA | CAGAACCGGCAACAAAACTC |
| Fbxw7 γ | CCTTGGGCAATGATGCTAAT | TTCCCGGTTTTGACATTTT |
| Primers for RT-PCR | | |
| Name | Forward sequences ( 5'-3') | Reverse Sequences ( 5'-3') |
| Fα | ATGATTCCCAGCAAGGACAG |  |
| Fβ | TGCTGAGGGAGGAATCTGTT |  |
| Fγ | GGACATTTGGTAGGGGAAGG |  |
| F1 | GAAGGAGGAAGGGAACCAAC |  |
| F2 | TCTCCCAAACCTGACTGTCC |  |
| F-ab | TCCACCTCCTGGGCTCAAGT |  |
| R2 |  | GTTGGTTCCCTTCCTCCTTC |
| cdc4R |  | GGCACGTCAGAAAAGGAAGA |
| R-ad |  | ACTTGAGCCCAGGAGGTGGA |
| Primers for detecting mutation and deletion in primary tumors | | |
| P1F/ R | ATGAATCAGGAACTGCTCTCTGT | AGGGAGCAATGAAATGAA |
| P2F/ R | ATTACATCTGTCCAGCCACC | CAAACCCTAAGAGTGGCA |
| P3F/ R | GAATAGTTAGTGGTTCTGATGA | CAGCACCAGCAGCTTGGT |
| GAPDH F/R | AGGTCGGAGTCAACGGATTTG | GTGATGGCATGGACTGTGGT |
| Primers for plasmid constructs | | |
| GL3F/R | GCAAAGCTTAAGCTTGAGTCTCCCAAACCTGACTG | GGACCATGGCCATGGTTCCAAAAGCCAGCTTGCTAC |
| GL3R2 |  | GGAGAATTCCCATGGTTCCAAAAGCCAGCTTGCTAC |
| Primers for real-time RT-PCR | | |
| LucF/ LucR | CAACTGCATAAGGCTATGAAGAGA | ATTTGTATTCAGCCCATATCGTTT |
| ReniF/ ReniR | GAGCATCAAGATAAGATCAAAGCA | CTTCACCTTTCTCTTTGAATGGTT |
