# Supplementary material for: Multiple Novel Alternative Splicing Forms of FBXW7α Have a Translational Modulatory Function and Show Specific Alteration in Human Cancer
Source: PLoS One. 2012 Nov 14;7(11):e49453. doi: 10.1371/journal.pone.0049453 (PMC3498124; doi:10.1371/journal.pone.0049453)
Supplement: Table S2 — The sequences of each FBXW7a 5′-UTR AS form. (DOC) [file pone.0049453.s005.doc]

**Table S2.** The sequences of each FBXW7a 5’-UTR AS form.

| **Sequences of 5’-UTR of Spli1**  ggaaactttacaaaaacaaaatccggagtctcccaaacctgactgtcccgggagaagtggccctggacgggcagaagccgcagcctgaaaagacccaggaagaggaaaagaggaggtaagcggggccgccgcctcccctcccctggcagcgcggaagagacccgggttgccgcctggtttagcgacacgagcaccgcttcttcctcagtaccgcgccggagccttccgcagctgccgcttcagtccgaaggaggaagggaaccaacccactttctcggcgccgcggctcttttctaaaagtgattacttccttaggatagattgccagaagtggagttactgggtcagagaatgtgaaaacctttgcatcttctgatagtctagccaaggtccaagaagtagcaagctggcttttggaa**ATG** |
| --- |
| **Sequences of 5’-UTR of Spli2**  ggaaactttacaaaaacaaaatccggagtctcccaaacctgactgtcccgggagaagtggccctggacgggcagaagccgcagcctgaaaagacccaggaagaggaaaagaggaggtaagcggggccgccgcctcccctcccctggcagcgcggaagagacccgggttgccgcctggtttagcgacacgagcaccgcttcttcctcagtaccgcgccggagccttccgcagctgccgcttcagtccgaaggaggaagggaaccaacccactttctcggcgccgcggctcttttctaaaagtaatgtgaaaacctttgcatcttctgatagtctagccaaggtccaagaagtagcaagctggcttttggaa**ATG** |
| **Sequences of 5’-UTR of Spli3**  ggaaactttacaaaaacaaaatccggagtctcccaaacctgactgtcccgggagaagtggccctggacgggcagaagccgcagcctgaaaagacccaggaagaggaaaagaggagtaccgcgccggagccttccgcagctgccgcttcagtccgaaggaggaagggaaccaacccactttctcggcgccgcggctcttttctaaaagtggtcttgctctgtcgtccaagctggagtgcggtcacagctcactgcagcctccacctcctgggctcaagtgatcttcccactttagcttcccaagtaggaagtaggtgggactacaggattacttccttaggatagattgccagaagtggagttactgggtcagagaatgtgaaaacctttgcatcttctgatagtctagccaaggtccaagaagtagcaagctggcttttggaa**ATG** |
| **Sequences of 5’-UTR of Spli4**  ggaaactttacaaaaacaaaatccggagtctcccaaacctgactgtcccgggagaagtggccctggacgggcagaagccgcagcctgaaaagacccaggaagaggaaaagaggagtaccgcgccggagccttccgcagctgccgcttcagtccgaaggaggaagggaaccaacccactttctcggcgccgcggctcttttctaaaagtgattacttccttaggatagattgccagaagtggagttactgggtcagagaatgtgaaaacctttgcatcttctgatagtctagccaaggtccaagaagtagcaagctggcttttggaa**ATG** |
| **Sequences of 5’-UTR of Spli5**  ggaaactttacaaaaacaaaatccggagtctcccaaacctgactgtcccgggagaagtggccctggacgggcagaagccgcagcctgaaaagacccaggaagaggaaaagaggagtaccgcgccggagccttccgcagctgccgcttcagtccgaaggaggaagggaaccaacccactttctcggcgccgcggctcttttctaaaagtaatgtgaaaacctttgcatcttctgatagtctagccaaggtccaagaagtagcaagctggcttttggaa**ATG** |
| **Sequences of 5’-UTR of Spli6**  ggaaactttacaaaaacaaaatccggagtctcccaaacctgactgtcccgggagaagtggccctggacgggcagaagccgcagcctgaaaagacccaggaagaggaaaagaggaggattacttccttaggatagattgccagaagtggagttactgggtcagagaatgtgaaaacctttgcatcttctgatagtctagccaaggtccaagaagtagcaagctggcttttggaa**ATG** |
| **Sequences of 5’-UTR of Spli7**  ggaaactttacaaaaacaaaatccggagtctcccaaacctgactgtcccgggagaagtggccctggacgggcagaagccgcagcctgaaaagacccaggaagaggaaaagaggagaatgtgaaaacctttgcatcttctgatagtctagccaaggtccaagaagtagcaagctggcttttggaa**ATG** |

The bold capital ‘ATG’ indicates the translational starting site.
